# A retrospective analysis of the prognostic value of nutritional-inflammatory markers for patients with cervical cancer

**DOI:** 10.7717/peerj.21273

**Published:** 2026-05-06

**Authors:** Ying Zhang, Wen Xing, Xiaoyi Liang, Yun Ma, Zhujuan Yang, Weipei Zhu

**Affiliations:** Department of Obstetrics and Gynecology, The Second Affiliated Hospital of Soochow University, Suzhou, Jiangsu, China

**Keywords:** PNI, NPS, Cervical cancer, Prognosis, Nutritional-inflammatory markers

## Abstract

**Background:**

Elevated inflammatory markers are consistently linked to poor outcomes in cancer, whereas favorable nutritional status correlates with improved survival. This retrospective study examined the interaction between nutritional-inflammatory indices and their impact on outcomes in patients with cervical cancer.

**Methods:**

Data from 465 cervical cancer patients treated at the Second Affiliated Hospital of Soochow University between January 2015 and June 2025 were retrospectively analyzed. The neutrophil-to-lymphocyte ratio (NLR), lymphocyte-to-monocyte ratio (LMR), Prognostic Nutritional Index (PNI), and Naples Prognostic Score (NPS) were calculated. Associations of these indices with overall survival (OS) and progression-free survival (PFS) were assessed using the Kaplan–Meier method and Cox regression models. Prognostic value was further evaluated through construction of a nomogram, with predictive accuracy validated using receiver operating characteristic (ROC) curves, decision curve analysis (DCA), and calibration analysis.

**Results:**

Significant differences between deceased patients and survivors were observed in body mass index (BMI), tumor burden, tumor markers, nutritional/inflammatory indices, pathological characteristics, and treatment status (all *p* < 0.05). Higher LMR, lower NLR, elevated PNI, and lower NPS were associated with improved OS and PFS (*p* < 0.001). Multivariate analysis identified BMI, PNI, Federation Internationale de Gynecologie et d’Obstetrique (FIGO) stage, differentiation, CA125, HPV infection, and targeted therapy as independent determinants of OS, while BMI, tumor volume, PNI, HPV infection, histology, differentiation, FIGO stage, and NPS independently predicted PFS. BMI exhibited a linear relationship with both OS and PFS, while PNI demonstrated a linear negative correlation with OS and a significant non-linear relationship with PFS. Nomograms incorporating eight variables for OS and six for PFS achieved AUC values ≥ 0.86 at 3-, 5-, and 10-year timepoints, demonstrating reliable discrimination, accurate calibration, and greater net clinical benefit compared with individual markers. Subgroup analyses consistently indicated protective effects of PNI (HR∼0.9) and low NPS (HR 0.3–0.9), though prognostic strength varied slightly according to clinical context and treatment approach.

**Conclusions:**

PNI was validated as a robust independent prognostic factor for both OS and PFS, while NPS demonstrated independent predictive value specifically for PFS. As easily obtainable nutritional-inflammatory indices, they complement conventional clinicopathological parameters. The integrated prognostic model established in this study shows exploratory potential in estimating OS and PFS. Pending future external validation, it may serve as an adjunctive reference for risk stratification and follow-up optimization.

## Introduction

Cervical cancer ranks among the most prevalent malignant tumors in women worldwide and continues to exhibit high incidence and mortality rates within the female reproductive system, representing a major threat to women’s health. Data from the World Health Organization indicate more than 600,000 newly diagnosed cases and approximately 340,000 deaths annually, with developing countries accounting for over 80% of the burden (Guo, Xu & Du, 2021). In China, both the incidence and mortality have shown a persistent upward trend, while the age of onset has shifted downward, highlighting its emergence as a pressing public health challenge. Despite advances in screening technologies and the integration of multimodal therapies, including surgery, radiotherapy, chemotherapy, and targeted therapy, marked heterogeneity persists in clinical outcomes, and the prognosis for patients with advanced, recurrent, or metastatic disease remains unsatisfactory, with limited improvement in the 5-year survival rate (Tshewang, Satiracoo & Lenbury, 2021). Accurate prognostic assessment is therefore essential for refining treatment strategies and improving survival outcomes. Conventional clinical prognostic factors primarily encompass clinicopathological characteristics such as Federation Internationale de Gynecologie et d’Obstetrique (FIGO) stage, pathological subtype, differentiation grade, lymph node involvement, and tumor marker levels (Zhou et al., 2021). While these variables provide partial insights into tumor aggressiveness and progression, they fall short of comprehensively capturing patients’ overall condition and treatment tolerance. With ongoing advances in tumor immunology and nutrition, increasing evidence indicates that nutritional status and systemic inflammatory responses exert significant influence on tumor initiation, progression, and prognosis (Huang et al., 2019). Chronic inflammation within the tumor microenvironment contributes to disease progression through mechanisms including accelerated angiogenesis, suppression of antitumor immunity, and enhanced tumor cell proliferation. Nevertheless, peripheral blood inflammatory markers fluctuate markedly with systemic immune and inflammatory status, limiting their utility as direct prognostic indicators (Ding et al., 2022). Consequently, composite indices that integrate immune and inflammatory parameters have been developed and broadly applied in prognostic evaluation of malignant tumors. Among them, the Naples Prognostic Score (NPS), which incorporates immune and nutritional variables, has demonstrated predictive value in female malignancies such as locally advanced cervical cancer and triple-negative breast cancer (Wang et al., 2023; Han et al., 2024). Malnutrition further aggravates immune dysfunction and reduces tolerance to therapy, thereby impairing treatment response and survival outcomes. Evidence indicates that the Prognostic Nutritional Index (PNI) serves as a reliable predictor of clinical prognosis in cancer patients (Yamamoto, Kawada & Obama, 2021; Zhou et al., 2023), with reduced PNI scores correlating with inferior overall survival (OS) and progression-free survival (PFS) in cervical cancer. On this basis, the present study conducted a retrospective analysis of clinical data from cervical cancer patients treated in this institution to assess the prognostic significance of PNI and NPS in this disease.

## Materials & Methods

### Study population

The study was carried out at the Second Affiliated Hospital of Soochow University between January 2015 and June 2025, enrolling 465 hospitalized gynecological patients with histopathologically confirmed cervical cancer in a retrospective design. Eligibility criteria included: (1) age ≥18 years; (2) diagnosis consistent with established clinical criteria for cervical cancer; and (3) patients who were newly diagnosed and received standard first-line treatment (including surgery, radiotherapy, chemotherapy, or targeted therapy) at our institution. Exclusion criteria comprised: (1) incomplete data for essential laboratory parameters; and (2) patients with recurrent disease at the time of admission or those who had received prior anticancer treatment. The investigation conformed to the ethical principles outlined in the Declaration of Helsinki (2013 revision) and was approved by the Ethics Committee of the Second Affiliated Hospital of Soochow University (approval number: JD-HG-2025-077). The requirement for informed consent was waived by the Ethics Committee because of the retrospective, anonymised nature of the analysis.

### Collection of clinical data and follow-up

The study included the following variables: demographic parameters (age and body mass index (BMI)); laboratory measurements (total cholesterol, albumin, neutrophil, lymphocyte, and monocyte counts, CA125, CEA, and SCC Ag); comorbidities (hypertension and diabetes); tumor characteristics comprising tumor size, histological subtype (squamous cell carcinoma or adenocarcinoma), HPV status (type 16, type 18, other types, or negative), lymph node involvement, FIGO stage (I–IV), degree of differentiation (poor, moderate, or well), and treatment modalities including surgery, radiotherapy, chemotherapy, and targeted therapy; as well as the interval from diagnosis to treatment initiation.

In this study, targeted therapy exclusively refers to bevacizumab, an anti-vascular endothelial growth factor (anti-VEGF) monoclonal antibody. It should be clearly clarified that the study cohort consists solely of newly diagnosed cervical cancer patients receiving first-line treatment (*i.e.,* primary treatment), and bevacizumab is not used as a first-line therapeutic agent. Instead, bevacizumab is only administered to patients in the cohort who develop disease persistence, recurrence, or metastasis during follow-up. Patients eligible for bevacizumab treatment met the following criteria: Eastern Cooperative Oncology Group (ECOG) performance status 0–1; adequate hematologic, renal, and hepatic function; no uncontrolled hypertension; no history of severe bleeding or gastrointestinal fistula. Strict adherence to these criteria was applied throughout the study to ensure the treatment protocol is explicit and reproducible.

Laboratory indicators and tumor-related parameters were obtained by qualified staff, with biochemical analyses performed using the same automated system. Laboratory physicians completed the operations, and pathologists provided the histopathological grading. Comorbidity information was derived from patients’ medical histories, while the time from diagnosis to treatment was retrieved from the discharge database of the Second Affiliated Hospital of Soochow University. Follow-up was conducted after discharge through outpatient visits or telephone interviews, focusing on 3-, 5-, and 10-year survival outcomes. The primary endpoints were OS and PFS. OS was defined as the interval from treatment initiation to death from any cause, with censoring at the last follow-up for patients lost to follow-up. PFS was defined as the interval from treatment initiation to the first radiologically confirmed tumor progression or death from any cause. Data description: Due to the retrospective design adopted, patients with missing values for key variables (including albumin, neutrophils, lymphocytes, and monocytes) were excluded from the study to ensure the accuracy of multivariate analysis and the construction of the nomogram.

### Calculation of nutritional and inflammatory indicators

**Figure 1:**
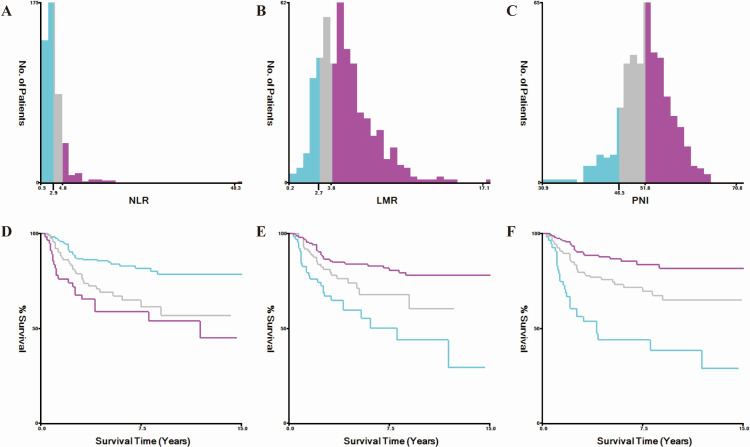
X-tile software was used to determine the optimal cut-off values for the neutrophil-to-lymphocyte ratio (NLR, A), lymphocyte-to-monocyte ratio (LMR, B), and prognostic nutritional index (PNI, C) based on overall survival outcomes. (D), (E), and (F) show the corresponding Kaplan–Meier overall survival curves for patients stratified by the optimal cut-off values of NLR, LMR, and PNI, respectively.

The neutrophil-to-lymphocyte ratio (NLR) and lymphocyte-to-monocyte ratio (LMR) were calculated as follows: NLR = neutrophil count/lymphocyte count and LMR = lymphocyte count/monocyte count. The prognostic nutritional index (PNI) was calculated using the formula: PNI = serum albumin (g/L) + 5 × total lymphocyte count (10^9^/L). Determination of Cut-off Values: The optimal cut-off values for PNI were determined using X-tile software based on survival outcomes. Patients were stratified into three groups: low (<46.5), moderate (46.5–51.8), and high (>51.8) (Fig. 1).

Definition of NPS: The NPS was determined based on four parameters: albumin, total cholesterol (TC), NLR, and LMR. A score of 1 was assigned for each of the following abnormal values: albumin < 40 g/L, TC ≤ 4.68 mmol/L, NLR ≥ 2.96, or LMR ≤ 4.44. Conversely, a score of 0 was assigned for values within the normal range (albumin ≥ 40 g/L, TC > 4.68 mmol/L, NLR < 2.96, and LMR > 4.44). The final NPS score ranged from 0 to 4. Patients were subsequently classified into the L-NPS group (score 0–2) or H-NPS group (score 3–4) (Han et al., 2024).

### Statistical analysis

In this study, baseline characteristics of cervical cancer patients were summarized, with stratification performed according to OS and PFS status. Non-normally distributed continuous variables were presented as median (interquartile range) and compared using the Wilcoxon rank-sum test, whereas categorical variables were expressed as frequency (percentage) and compared with the chi-square test. Survival distributions of NLR, LMR, PNI, and NPS across groups were illustrated using Kaplan–Meier curves, and intergroup differences were examined by the Log-rank test. Associations between each nutritional and inflammatory indicator and prognosis were quantified through hazard ratios (HR) derived from univariate and multivariate Cox proportional hazards models. To construct the multivariable models, variables demonstrating statistical significance (*p* < 0.05) in the univariate analysis were considered. To mitigate the risk of multicollinearity given the shared components among nutritional-inflammatory indices, individual foundational parameters (*e.g.*, absolute neutrophil counts, lymphocyte counts, and serum albumin) were a priori excluded from the multivariable analyses, retaining only their respective composite indices. Furthermore, potential multicollinearity was assessed using the Variance Inflation Factor (VIF). All VIF values were confirmed to be <5, indicating no severe collinearity and ensuring model stability. To assess potential dose–response relationships between selected indicators and survival, a restricted cubic spline (RCS) model was applied. Prognostic factors with statistical significance in multivariate analysis were incorporated into a Nomogram to estimate 3-, 5-, and 10-year survival probabilities. Patients were classified into two risk groups according to the median risk score, and their outcomes were further examined using Kaplan–Meier curves. The predictive accuracy of the model was evaluated through receiver operating characteristic (ROC) curves, decision curve analysis (DCA), and calibration plots. Internal validation was performed using 1,000 bootstrap resamples to assess the model’s stability and prevent overfitting. ROC curves of individual indicators were additionally constructed to compare prognostic discrimination, and stratified analyses were performed to determine the prognostic relevance of nutritional and inflammatory indicators in subgroups with distinct clinical features. To address the potential survival time bias caused by the inconsistent time intervals between diagnosis and treatment, the researchers conducted a sensitivity analysis by resetting the starting points of the OS and PFS times to the date of diagnosis. All statistical analyses were carried out in R software (version 4.3.0) and STATA 17.0 (64-bit), with α set at 0.05 for two-tailed tests.

**Table 1:** Baseline demographic and clinical characteristics of patients with cervical cancer. Baseline characteristics of 465 cervical-cancer patients stratified by overall survival (OS) and progression-free survival (PFS) status: comparison of deceased vs surviving groups for demographic, laboratory, pathological and treatment variables.

**Variables**	**Total (*N* = 465)**	**OS**		***p***	**PFS**		***p***
		**Survival (*N* = 369)**	**Dead (*N* = 96)**		**Survival (*N* = 344)**	**Dead (*N* = 121)**	
Age, years	56.00 (49.50, 64.50)	56.00 (50.00, 64.00)	54.50 (48.00, 69.75)	0.563	56.00 (50.00, 64.00)	55.00 (48.50, 69.00)	0.531
BMI	22.50 (21.00, 24.43)	22.70 (21.00, 24.77)	21.48 (20.32, 23.00)	<0.001	22.79 (21.09, 24.76)	21.51 (20.06, 23.13)	<0.001
Tumor_size	3.00 (2.00, 5.00)	3.00 (1.50, 4.00)	5.00 (3.50, 6.00)	<0.001	3.00 (1.07, 4.00)	5.00 (3.50, 6.00)	<0.001
Diagnosis_to_Treatment _Interval	20.00 (13.00, 32.00)	21.00 (14.00, 33.00)	17.00 (9.00, 29.75)	0.007	20.00 (14.00, 33.00)	18.00 (10.00, 30.00)	0.065
SCC_Ag	2.00 (0.90, 6.06)	1.74 (0.84, 4.56)	5.72 (1.70, 11.50)	<0.001	1.61 (0.82, 4.04)	5.72 (1.70, 12.31)	<0.001
CEA	2.36 (1.42, 3.76)	2.30 (1.41, 3.48)	3.13 (1.53, 4.96)	0.020	2.24 (1.41, 3.46)	3.00 (1.54, 4.88)	0.015
CA125	19.15 (13.00, 31.24)	18.20 (12.10, 26.02)	30.27 (18.70, 55.63)	<0.001	17.94 (12.00, 25.34)	29.00 (17.16, 54.52)	<0.001
Albumin	44.40 (41.52, 46.70)	44.90 (42.40, 47.00)	41.90 (37.92, 44.88)	<0.001	44.95 (42.50, 47.08)	42.40 (38.40, 45.20)	<0.001
Total_Cholesterol	4.77 (4.20, 5.36)	4.77 (4.24, 5.36)	4.77 (3.99, 5.29)	0.275	4.78 (4.24, 5.36)	4.77 (4.09, 5.33)	0.530
Lymphocyte	1.60 (1.20, 1.90)	1.60 (1.20, 1.90)	1.54 (1.12, 1.90)	0.632	1.60 (1.20, 1.90)	1.60 (1.20, 1.90)	0.904
Neutrophil	4.00 (2.94, 5.10)	3.80 (2.90, 4.80)	4.90 (3.23, 6.10)	<0.001	3.80 (2.82, 4.80)	4.70 (3.36, 6.10)	<0.001
Monocyte	0.35 (0.27, 0.40)	0.30 (0.27, 0.40)	0.40 (0.28, 0.52)	0.003	0.30 (0.26, 0.40)	0.40 (0.30, 0.54)	<0.001
NLR	2.50 (1.80, 3.80)	2.30 (1.70, 3.40)	3.30 (2.10, 4.47)	<0.001	2.30 (1.70, 3.30)	3.30 (2.05, 4.40)	<0.001
LMR	4.50 (3.30, 6.30)	4.80 (3.45, 6.30)	3.80 (2.60, 5.70)	0.001	4.80 (3.52, 6.50)	3.60 (2.60, 5.60)	<0.001
PNI	52.20 (48.75, 55.25)	52.70 (49.55, 55.55)	49.65 (46.23, 52.77)	<0.001	52.75 (49.60, 55.50)	50.30 (46.50, 53.25)	<0.001
Diabetes, n(%)				0.126			0.067
No	429 (92.3%)	344 (93.2%)	85 (88.5%)		322 (93.6%)	107 (88.4%)	
Yes	36 (7.7%)	25 (6.8%)	11 (11.5%)		22 (6.4%)	14 (11.6%)	
Hypertension, n(%)				0.491			0.170
No	384 (82.6%)	307 (83.2%)	77 (80.2%)		289 (84.0%)	95 (78.5%)	
Yes	81 (17.4%)	62 (16.8%)	19 (19.8%)		55 (16.0%)	26 (21.5%)	
HPV_Infection, n(%)				0.001			0.003
16	267 (57.4%)	219 (59.3%)	48 (50.0%)		202 (58.7%)	65 (53.7%)	
18	33 (7.1%)	19 (5.1%)	14 (14.6%)		19 (5.5%)	14 (11.6%)	
Others	92 (19.8%)	79 (21.4%)	13 (13.5%)		77 (22.4%)	15 (12.4%)	
Negative	73 (15.7%)	52 (14.1%)	21 (21.9%)		46 (13.4%)	27 (22.3%)	
Histology_type, n(%)				<0.001			0.003
Squamous cell carcinoma	411 (88.4%)	336 (91.1%)	75 (78.1%)		313 (91.0%)	98 (81.0%)	
Adenocarcinoma	54 (11.6%)	33 (8.9%)	21 (21.9%)		31 (9.0%)	23 (19.0%)	
Degree_of_differentiation, n(%)				<0.001			<0.001
Low-differentiated	182 (39.1%)	111 (30.1%)	71 (74.0%)		94 (27.3%)	88 (72.7%)	
Medium-differentiated	230 (49.5%)	207 (56.1%)	23 (24.0%)		200 (58.1%)	30 (24.8%)	
High-differentiated	53 (11.4%)	51 (13.8%)	2 (2.1%)		50 (14.5%)	3 (2.5%)	
FIGO_Stage, n(%)				<0.001			<0.001
Grade I	199 (42.8%)	195 (52.8%)	4 (4.2%)		191 (55.5%)	8 (6.6%)	
Grade II	101 (21.7%)	83 (22.5%)	18 (18.8%)		80 (23.3%)	21 (17.4%)	
Grade III	119 (25.6%)	76 (20.6%)	43 (44.8%)		63 (18.3%)	56 (46.3%)	
Grade IV	46 (9.9%)	15 (4.1%)	31 (32.3%)		10 (2.9%)	36 (29.8%)	
Lymph_node_metastasis, n(%)				<0.001			<0.001
No	331 (71.2%)	296 (80.2%)	35 (36.5%)		286 (83.1%)	45 (37.2%)	
Yes	134 (28.8%)	73 (19.8%)	61 (63.5%)		58 (16.9%)	76 (62.8%)	
Radiotherapy, n(%)				<0.001			<0.001
No	124 (26.7%)	118 (32.0%)	6 (6.2%)		115 (33.4%)	9 (7.4%)	
Yes	341 (73.3%)	251 (68.0%)	90 (93.8%)		229 (66.6%)	112 (92.6%)	
Chemotherapy, n(%)				<0.001			<0.001
No	146 (31.4%)	140 (37.9%)	6 (6.2%)		136 (39.5%)	10 (8.3%)	
Yes	319 (68.6%)	229 (62.1%)	90 (93.8%)		208 (60.5%)	111 (91.7%)	
Surgery, n(%)				<0.001			<0.001
No	145 (31.2%)	81 (22.0%)	64 (66.7%)		68 (19.8%)	77 (63.6%)	
Yes	320 (68.8%)	288 (78.0%)	32 (33.3%)		276 (80.2%)	44 (36.4%)	
Targeted_therapy, n(%)				0.055			<0.001
No	405 (87.1%)	327 (88.6%)	78 (81.2%)		316 (91.9%)	89 (73.6%)	
Yes	60 (12.9%)	42 (11.4%)	18 (18.8%)		28 (8.1%)	32 (26.4%)	
NLR, n(%)				<0.001			<0.001
<2.9	272 (58.5%)	232 (62.9%)	40 (41.7%)		221 (64.2%)	51 (42.1%)	
2.9–4.8	136 (29.2%)	102 (27.6%)	34 (35.4%)		92 (26.7%)	44 (36.4%)	
>4.8	57 (12.3%)	35 (9.5%)	22 (22.9%)		31 (9.0%)	26 (21.5%)	
LMR, n(%)				<0.001			<0.001
<2.7	62 (13.3%)	37 (10.0%)	25 (26.0%)		31 (9.0%)	31 (25.6%)	
2.7–3.8	109 (23.4%)	83 (22.5%)	26 (27.1%)		74 (21.5%)	35 (28.9%)	
>3.8	294 (63.2%)	249 (67.5%)	45 (46.9%)		239 (69.5%)	55 (45.5%)	
PNI, n(%)				<0.001			<0.001
<46.5	52 (11.2%)	26 (7.0%)	26 (27.1%)		23 (6.7%)	29 (24.0%)	
46.5–51.8	176 (37.8%)	135 (36.6%)	41 (42.7%)		125 (36.3%)	51 (42.1%)	
>51.8	237 (51.0%)	208 (56.4%)	29 (30.2%)		196 (57.0%)	41 (33.9%)	
NPS, n(%)				<0.001			<0.001
L_NPS	251 (54.0%)	217 (58.8%)	34 (35.4%)		207 (60.2%)	44 (36.4%)	
H_NPS	214 (46.0%)	152 (41.2%)	62 (64.6%)		137 (39.8%)	77 (63.6%)	

**Notes.**

Abbreviations: Percentages may not total 100 because of rounding.

## Results

### Demographic and clinical characteristics of cervical cancer patients

A total of 465 patients with cervical cancer were enrolled, and stratified analyses were performed according to OS and PFS status (Table 1). Significant differences in multiple clinical parameters were observed between deceased and surviving patients. Regarding general features, the deceased group exhibited lower BMI, larger tumor volumes, and shorter intervals from diagnosis to treatment, all reaching statistical significance (*p* < 0.05). Tumor marker assessment revealed higher concentrations of SCC-Ag, CEA, and CA125 in deceased patients (*p* < 0.05). Evaluation of nutritional and inflammatory indices demonstrated impaired nutritional condition and heightened inflammatory activity, characterized by reduced serum albumin, elevated neutrophil counts, markedly higher NLR, and significantly lower LMR and PNI (*p* < 0.05). Pathological analyses further indicated a higher prevalence of HPV-18 infection, adenocarcinoma histology, poor differentiation, advanced FIGO stage, and lymph node metastasis in the deceased cohort (*p* < 0.05). Moreover, treatment modality was strongly associated with prognosis, as patients who did not undergo surgery, radiotherapy, or chemotherapy exhibited markedly worse survival outcomes (*p* < 0.05). In terms of follow-up and survival outcomes, the mean follow-up time for the entire cohort was 55.53 months, with a median follow-up time of 44.0 months. During the observation period, 96 (20.6%) patients died, and 369 (79.4%) were censored for OS. Regarding PFS, the mean time was 52.35 months, while the median follow-up time was 40.0 months; 121 (26.0%) events (tumor progression or death) were documented, while 344 (74.0%) observations were censored.

### Identification of prognostic factors for os and pfs in cervical cancer patients

**Figure 2:**
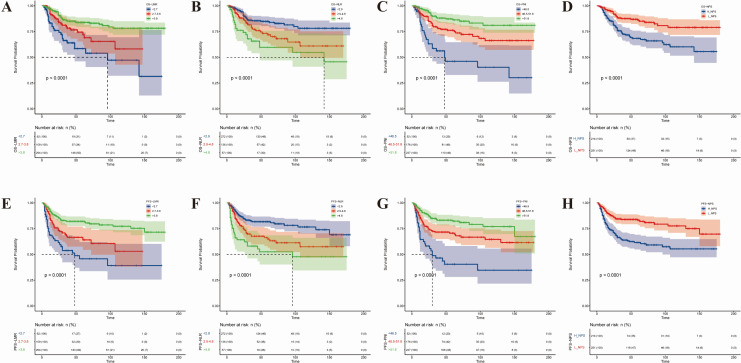
Kaplan–Meier survival curves evaluated the effects of LMR, NLR, PNI, NPS on OS and PFS. Kaplan–Meier survival curves by nutritional–inflammatory biomarkers in cervical-cancer patients. (A) OS by LMR groups. (B) OS by NLR groups. (C) OS by PNI groups; (D) OS by NPS groups. (E) PFS by LMR groups. (F) PFS by NLR groups; (G) PFS by PNI groups. (H) PFS by NPS groups.

Kaplan–Meier survival analysis was applied to examine the association of four nutritional and inflammatory indicators (LMR, NLR, PNI, and NPS) with OS and PFS (Fig. 2). All indicators demonstrated strong statistical significance in predicting survival outcomes (*p* < 0.001). Elevated LMR was correlated with reduced mortality risk and prolonged OS and PFS (Figs. 2A and 2E), reflecting the protective effect of robust immune function on patient outcomes. Conversely, a lower NLR was linked to improved prognosis (Figs. 2B and 2F), consistent with the interpretation that diminished systemic inflammation favors disease control. Moreover, higher PNI and lower NPS were both significantly related to reduced mortality and extended OS and PFS (Figs. 2C, 2D, 2G, and 2H), highlighting the prognostic relevance of nutritional status in cervical cancer.

**Table 2:** Univariate and multivariate analyses of overall survival (OS) in patients with cervical cancer. Univariate and multivariate Cox regression results for overall survival (OS) in 465 cervical cancer patients: hazard ratios (HRs) with 95% CIs and *p*-values for clinical, laboratory and therapeutic variables.

**Characteristic**	**Univariate analysis**			**Multivariate analysis**	
	***P***	**HR (95% CI)**		***P***	**HR (95% CI)**
Age, years	0.681	1.00 (0.99, 1.02)			
BMI	<0.001	0.87 (0.81, 0.94)		0.005	0.90 (0.84, 0.97)
Tumor_size	<0.001	1.61 (1.46, 1.78)		0.053	1.14 (0.99, 1.31)
Diagnosis_to_Treatment_Interval	0.746	1.00 (1.00, 1.00)		-	-
SCC_Ag	<0.001	1.04 (1.02, 1.05)		0.151	1.02 (0.99, 1.04)
CEA	<0.001	1.03 (1.01, 1.04)		0.072	1.02 (0.99, 1.04)
CA125	<0.001	1.01 (1.01, 1.01)		<0.001	1.02 (1.01, 1.02)
Albumin	<0.001	0.85 (0.82, 0.89)		-	-
Total_Cholesterol	0.186	0.87 (0.70, 1.07)		-	-
Lymphocyte	0.444	0.86 (0.59, 1.26)		-	-
Neutrophil	<0.001	1.21 (1.13, 1.29)		-	-
Monocyte	0.028	1.28 (1.03, 1.59)		-	-
NLR	<0.001	1.15 (1.10, 1.20)		-	-
LMR	<0.001	0.84 (0.76, 0.93)		-	-
PNI	<0.001	0.89 (0.86, 0.92)		-	-
Diabetes, n(%)					
No		Reference			
Yes	0.143	1.60 (0.85, 3.00)		-	-
Hypertension, n(%)					
No		Reference			
Yes	0.438	1.22 (0.74, 2.02)		-	-
HPV_Infection, n(%)					
16		Reference			Reference
18	<0.001	2.97 (1.63, 5.38)		<0.001	4.26 (1.87, 9.69)
Others	0.582	0.84 (0.46, 1.55)		0.345	1.37 (0.71, 2.63)
Negative	0.012	0.74 (0.66, 0.88)		0.706	1.12 (0.62, 2.02)
Histology_type, n(%)					
Squamous cell carcinoma		Reference			Reference
Adenocarcinoma	<0.001	2.68 (1.65, 4.36)		0.172	1.64 (0.81, 3.34)
Degree_of_differentiation, n(%)					
Low-differentiated		Reference			Reference
Medium-differentiated	<0.001	0.21 (0.13, 0.34)		0.004	0.45 (0.26, 0.77)
High-differentiated	<0.001	0.08 (0.02, 0.32)		0.136	0.32 (0.07,1.43)
FIGO_Stage, n(%)					
Grade I		Reference			Reference
Grade II	<0.001	2.48 (2.87, 5.04)		0.004	2.43 (1.57, 4.97)
Grade III	<0.001	3.21 (2.61, 6.10)		<0.001	3.17 (2.69, 6.12)
Grade IV	<0.001	3.71 (2.80, 7.31)		<0.001	3.71 (2.92, 6.75)
Lymph_node_metastasis, n(%)					
No		Reference			Reference
Yes	<0.001	5.81 (3.83, 8.82)		0.530	1.23 (0.65, 2.35)
Radiotherapy, n(%)					
No		Reference			Reference
Yes	<.001	5.81 (2.54, 13.29)		0.395	1.52 (0.58, 3.98)
Chemotherapy, n(%)					
No		Reference			Reference
Yes	<.001	7.60 (3.33, 17.38)		0.833	0.90 (0.34, 2.39)
Surgery, n(%)					
No		Reference			Reference
Yes	<0.001	0.17 (0.11, 0.27)		0.390	0.79 (0.47, 1.35)
Targeted_therapy, n(%)					
No		Reference			Reference
Yes	0.004	2.12 (1.26, 3.56)		0.007	0.39 (0.20, 0.78)
NLR, n(%)					
<2.9		Reference			Reference
2.9–4.8	0.009	1.84 (1.17, 2.91)		0.936	0.97 (0.5, 1.75)
>4.8	<0.001	3.05 (1.81, 5.13)		0.308	0.66 (0.30, 1.45)
LMR, n(%)					
<2.7		Reference			Reference
2.7–3.8	0.041	0.56 (0.33, 0.98)		0.202	0.64 (0.32, 1.27)
>3.8	<0.001	0.30 (0.18, 0.49)		0.444	0.76 (0.38, 1.51)
PNI, n(%)					
<46.5		Reference			Reference
46.5–51.8	<0.001	0.39 (0.24, 0.64)		0.049	0.61 (0.34, 0.99)
>51.8	<0.001	0.20 (0.12, 0.33)		0.003	0.43 (0.21, 0.87)
NPS, n(%)					
L_NPS		Reference			Reference
H_NPS	<0.001	1.42 (1.18, 2.64)		0.907	1.04 (0.52, 2.06)

**Table 3:** Univariate and multivariate analyses of progression-free survival (PFS) in patients with cervical cancer. Univariate and multivariate Cox regression results for progression-free survival (PFS) in 465 cervical-cancer patients: hazard ratios (HRs) with 95% CIs and *p*-values for clinical, laboratory and therapeutic variables.

**Characteristic**	**Univariate analysis**			**Multivariate analysis**	
	***P***	**HR (95% CI)**		***P***	**HR (95% CI)**
Age, years	0.580	1.00 (0.99, 1.02)		–	–
BMI	<0.001	0.88 (0.82, 0.94)		0.002	0.90 (0.85, 0.96)
Tumor_size	<0.001	1.57 (1.44, 1.71)		0.016	1.17 (1.03, 1.32)
Diagnosis_to_Treatment_Interval	0.720	1.00 (1.00, 1.00)		–	–
SCC_Ag	<0.001	1.04 (1.03, 1.05)		0.159	1.01 (0.99, 1.03)
CEA	<0.001	1.02 (1.01, 1.04)		0.891	0.99 (0.97, 1.02)
CA125	<0.001	1.01 (1.01, 1.01)		0.172	0.99 (0.99, 1.00)
Albumin	<0.001	0.86 (0.83, 0.90)		–	–
Total_Cholesterol	0.490	0.94 (0.78, 1.13)		–	–
Lymphocyte	0.714	0.94 (0.67, 1.32)		–	–
Neutrophil	<0.001	1.22 (1.15, 1.29)		–	–
Monocyte	00.015	1.27 (1.05, 1.54)		–	–
NLR	<0.001	1.12 (1.08, 1.15)		–	–
LMR	<0.001	0.83 (0.76, 0.91)		–	–
PNI	<0.001	0.90 (0.87, 0.94)		–	–
Diabetes, n(%)					
No		Reference			
Yes	0.057	1.72 (0.98, 3.00)		–	–
Hypertension, n(%)					
No		Reference			
Yes	0.129	1.40 (0.91, 2.16)		–	–
HPV_Infection, n(%)					
16		Reference			Reference
18	0.007	2.20 (1.24, 3.92)		0.045	1.93 (1.28, 4.25)
Others	0.210	0.70 (0.40, 1.22)		0.906	1.04 (0.57, 1.88)
Negative	0.014	1.76 (1.12, 2.76)		0.909	1.03 (0.63, 1.70)
Histology_type, n(%)					
Squamous cell carcinoma		Reference			Reference
Adenocarcinoma	<0.001	2.29 (1.45, 3.61)		0.013	2.27 (1.19, 4.32)
Degree_of_differentiation, n(%)					
Low-differentiated		Reference			Reference
Medium-differentiated	<0.001	0.20 (0.13, 0.31)		<0.001	0.40 (0.25, 0.65)
High-differentiated	<0.001	0.09 (0.03, 0.28)		0.085	0.34 (0.10, 1.16)
FIGO_Stage, n(%)					
Grade I		Reference			Reference
Grade II	<0.001	1.26 (2.33, 3.87)		0.012	3.13 (1.67, 5.27)
Grade III	<0.001	2.76 (2.03, 4.99)		0.002	3.34 (2.29, 5.12)
Grade IV	<0.001	3.84 (2.59, 6.62)		<0.001	3.71 (2.73, 6.12)
Lymph_node_metastasis, n(%)					
No		Reference			Reference
Yes	<0.001	5.98 (4.12, 8.66)		0.540	1.20 (0.67, 2.12)
Radiotherapy, n(%)					
No		Reference			Reference
Yes	<0.001	5.00 (2.53, 9.86)		0.488	1.35 (0.58, 3.12)
Chemotherapy, n(%)					
No		Reference			Reference
Yes	<0.001	5.94 (3.11, 11.34)		0.744	1.24 (0.71, 2.67)
Surgery, n(%)					
No		Reference			Reference
Yes	<0.001	0.19 (0.13, 0.28)		0.674	0.90 (0.56, 1.46)
Targeted_therapy, n(%)					
No		Reference			Reference
Yes	<0.001	3.56 (2.36, 5.38)		0.909	1.03 (0.61, 1.73)
NLR, n(%)					
<2.9		Reference			Reference
2.9–4.8	0.002	1.90 (1.27, 2.84)		0.906	0.97 (0.58, 1.62)
>4.8	<0.001	2.97 (1.85, 4.76)		0.411	0.73 (0.35, 1.54)
LMR, n(%)					
<2.7		Reference			Reference
2.7–3.8	0.028	0.58 (0.36, 0.94)		0.151	0.65 (0.36, 1.17)
>3.8	<0.001	0.28 (0.18, 0.44)		0.204	0.67 (0.36, 1.25)
PNI, n(%)					
<46.5		Reference			Reference
46.5–51.8	<0.001	0.44 (0.28, 0.69)		0.032	0.72 (0.46, 0.91)
>51.8	<0.001	0.24 (0.15, 0.39)		0.024	0.61 (0.24, 0.83)
NPS, n(%)					
L_NPS		Reference			Reference
H_NPS	<0.001	1.43 (1.29, 2.62)		0.036	1.27 (1.13, 1.97)

Univariate and multivariate Cox regression analyses were performed to identify prognostic factors for OS and PFS. For OS, univariate analysis indicated significant associations with multiple clinical and nutritional parameters (Table 2). In the multivariate analysis, BMI (HR = 0.90, 95% CI [0.84–0.97], *p* = 0.005), CA125 (HR = 1.02, 95% CI [1.01–1.02], *p* < 0.001), HPV-18 infection (HR = 4.26, 95% CI [1.87–9.69], *p* < 0.001), moderate differentiation (HR = 0.45, 95% CI [0.25–0.77], *p* = 0.024), advanced FIGO stage (Grade II: HR = 2.43; Grade III: HR = 3.17; Grade IV: HR = 3.71, all *p* < 0.01), and targeted therapy (HR = 0.39, 95% CI [0.20–0.78], *p* = 0.007) were identified as independent prognostic factors. Notably, high PNI remained an independent protective factor (HR = 0.43, 95% CI [0.21–0.87], *p* = 0.003), whereas NPS did not reach statistical significance in the multivariate model. For PFS, the multivariate analysis yielded distinct results (Table 3). Independent prognostic factors included BMI, tumor size, HPV-18 infection, adenocarcinoma histology, differentiation grade, and FIGO stage. Importantly, unlike in the OS model, both PNI (HR = 0.61, 95% CI [0.24–0.83], *p* = 0.024) and NPS (HR = 1.27, 95% CI [1.13–1.97], *p* = 0.036) demonstrated independent prognostic value for PFS. Furthermore, a sensitivity analysis was performed using the date of diagnosis as the starting point for OS and PFS to rigorously account for potential immortal time bias. Consistent with the primary analysis, the multivariate models confirmed that high PNI remained an independent protective factor for both OS (Table S1) and PFS (Table S2), while NPS maintained its independent prognostic value specifically for PFS (Table S2). These results demonstrate the robustness of the prognostic models regardless of the selected time origin.

### Relationship between BMI, PNI and survival prognosis of cervical cancer patients

**Figure 3:**
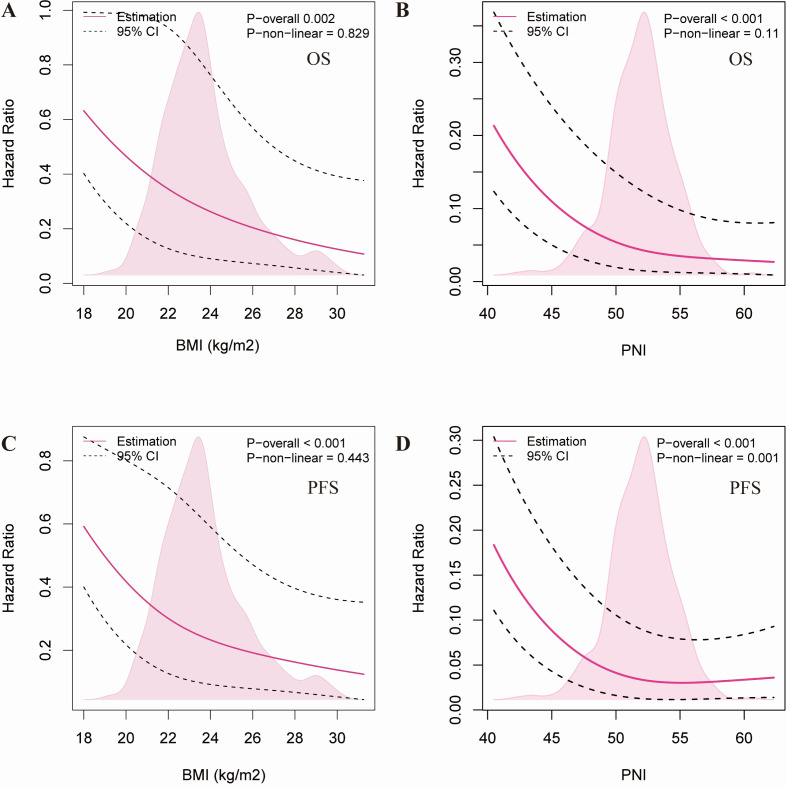
RCS diagram of the relationship between BMI and PNI and overall survival (OS) as well as progression-free survival (PFS) in cervical cancer patients. Restricted cubic spline plots illustrating non-linear relationships between BMI and PNI with overall survival (OS) and progression-free survival (PFS) in cervical cancer patients; reference = median (BMI 23 kg/m^2^; PNI 48), solid lines = HRs, shaded = 95% CI. (A) BMI *versus* OS. (B) PNI *versus* OS. (C) BMI *versus* PFS. (D) PNI *versus* PFS.

Variables identified as significant in univariate and multivariate Cox regression analyses were further examined using Restricted Cubic Spline (RCS) models to evaluate the dose–response association of BMI and PNI with survival outcomes (Fig. 3). For OS: BMI displayed an approximately linear inverse association with mortality risk (P-overall = 0.002, P-non-linear = 0.829), indicating that higher BMI was associated with better survival outcomes within the observed range (Fig. 3A). Similarly, PNI exhibited a linear negative correlation with OS (P-overall < 0.001, P-non-linear = 0.11), where elevated PNI levels consistently corresponded to a reduced risk of death (Fig. 3B). For PFS: Consistent with the OS results, BMI showed a linear protective effect against disease progression (P-overall < 0.001, P-non-linear = 0.443) (Fig. 3C). In contrast, PNI demonstrated a significant non-linear relationship with PFS (P-overall < 0.001, P-non-linear = 0.001). As shown in Fig. 3D, the risk of progression or death increased sharply as PNI levels dropped below a certain threshold, suggesting a potential saturation effect at higher levels. Overall, these findings suggest that maintaining an appropriate BMI and adequate nutritional status (reflected by high PNI) is essential for improving survival prognosis in cervical cancer.

### Construction and evaluation of prognostic nomogram for cervical cancer patients based on selected variables

**Figure 4:**
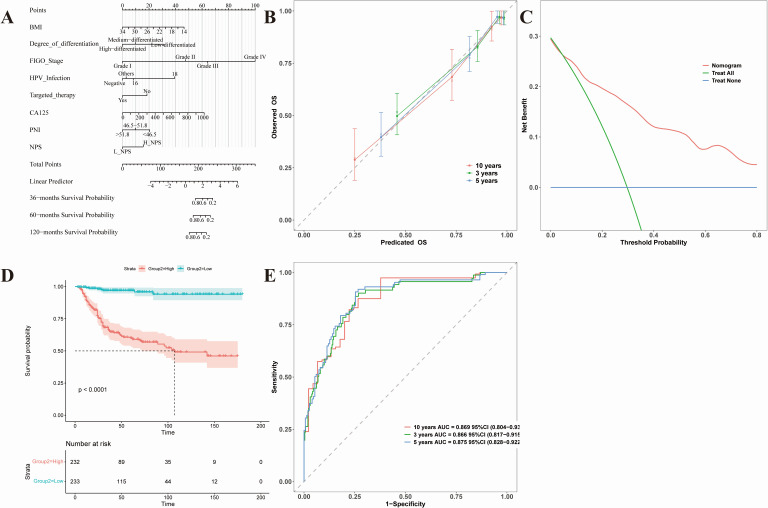
Nomogram prediction model for OS in cervical cancer patients. Nomogram-based OS model (BMI, differentiation, FIGO stage, HPV, targeted therapy, CA125, PNI, NPS) and its validation. (A) Nomogram for 3-, 5-, 10-year survival probabilities. (B) Calibration curves. (C) Decision-curve analysis. (D) Kaplan–Meier risk-stratified curves (*p* < 0.0001). (E) Time-dependent ROC (AUC 0.866–0.889).

**Figure 5:**
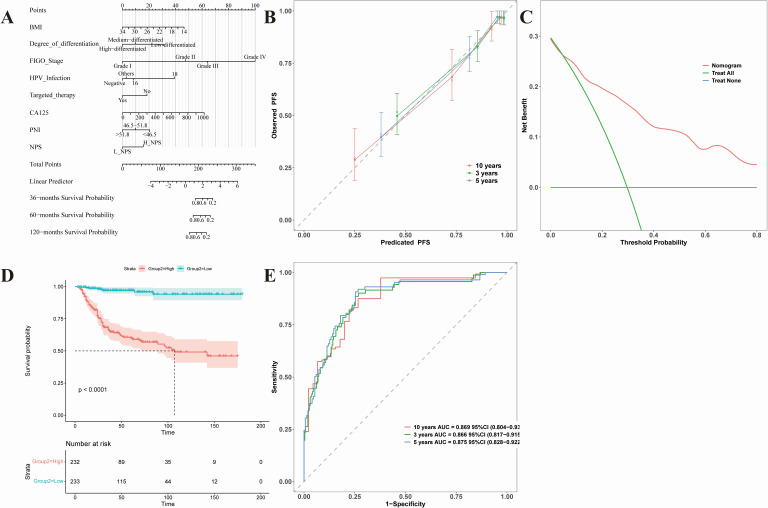
Nomogram prediction model for PFS in cervical cancer patients. Nomogram-based PFS model (BMI, histological type, differentiation, FIGO stage, PNI, NPS) and its validation. (A) Nomogram for 3-, 5-, 10-year progression-free probabilities. (B) Calibration curves. (C) Decision-curve analysis. (D) Kaplan–Meier risk-stratified curves (*p* < 0.0001). (E) Time-dependent ROC (AUC 0.866–0.875).

In this study, univariate and multivariate Cox regression analyses were applied to identify statistically significant prognostic variables, and predictive models for OS and PFS were subsequently established and rigorously validated through multiple approaches. For the OS model (Fig. 4), eight determinants—BMI, tumor differentiation, FIGO stage, HPV infection status, targeted therapy status, CA125 concentration, PNI, and NPS—were incorporated into a Nomogram framework (Fig. 4A), providing a practical visualization tool capable of estimating 3-, 5-, and 10-year survival probabilities with high precision. The validation results indicated that the calibration curve demonstrated strong concordance between predicted and observed outcomes, particularly at medium- and long-term follow-up (Fig. 4B). Decision curve analysis further confirmed meaningful clinical net benefit across a broad spectrum of threshold probabilities (Fig. 4C). Stratification of patients into high- and low-risk cohorts using the median risk score revealed a marked survival difference by Kaplan–Meier analysis (*p* < 0.0001) (Fig. 4D). Time-dependent ROC analysis showed that the AUC values at 3, 5, and 10 years reached 0.866, 0.875, and 0.889, respectively, reflecting strong discriminative capacity (Fig. 4E). Comparable performance was observed for the PFS prediction model (Fig. 5), which incorporated six variables: BMI, histological type, differentiation grade, FIGO stage, PNI, and NPS (Fig. 5A). The calibration curve exhibited robust agreement with observed outcomes (Fig. 5B), and decision curve analysis verified its clinical applicability (Fig. 5C). Risk stratification again revealed significant separation of PFS between high- and low-risk groups (*p* < 0.0001) (Fig. 5D). ROC analysis confirmed AUC values consistently above 0.86 at 3, 5, and 10 years (Fig. 5E).

### Comparison of diagnostic efficacy between single variables and comprehensive models in cervical cancer OS and PFS

**Figure 6:**
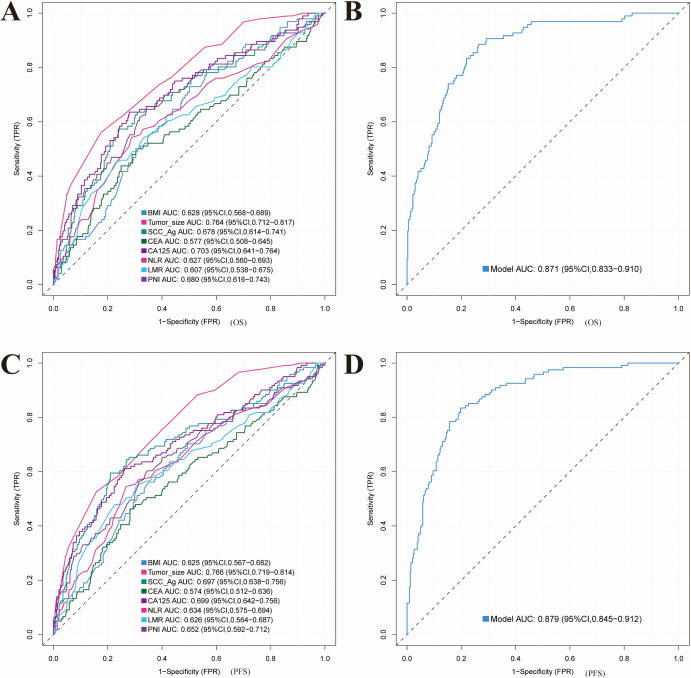
ROC curves of the diagnostic nomogram model for predicting overall survival (OS) and progression-free survival (PFS) in cervical cancer patients. ROC comparison of single biomarkers *versus* the integrated model for OS and PFS. (A) Individual ROC curves for OS: tumour size, CA125, NLR, PNI and others. (B) Combined model ROC for OS: AUC = 0.871 (95% CI [0.833–0.910]). (C) Individual ROC curves for PFS: tumour size, SCC-Ag, CA125, PNI and others. (D) Combined model ROC for PFS: AUC = 0.879 (95% CI [0.845–0.912]).

In this study, ROC curve analysis was applied to assess the diagnostic and prognostic performance of individual variables and integrated models for OS and PFS in cervical cancer (Fig. 6). For OS, variables including tumor size, CA125, NLR, and PNI demonstrated measurable predictive capacity (Fig. 6A). Tumor size yielded an AUC of 0.764 (95% CI [0.712–0.817]), while CA125 showed an AUC of 0.703 (95% CI [0.641–0.764]). By contrast, the comprehensive model achieved a markedly higher AUC of 0.871 (95% CI [0.833–0.910]), reflecting superior sensitivity and specificity in OS prediction (Fig. 6B). This enhanced performance stems from the model’s ability to synthesize clinical features with nutritional and inflammatory indicators, thereby improving predictive precision. In the evaluation of PFS, tumor size, SCC-Ag, CA125, and PNI also exhibited predictive value (Fig. 6C). Tumor size produced an AUC of 0.766 (95% CI [0.719–0.814]), whereas SCC-Ag reached 0.697 (95% CI [0.638–0.756]). The integrated model further improved prediction, with an AUC of 0.879 (95% CI [0.845–0.912]), confirming its superiority for PFS assessment (Fig. 6D). Unlike single-variable approaches, the comprehensive model incorporates both independent contributions and their interactions, thereby yielding a more precise prognostic evaluation. In summary, although tumor size, CA125, and PNI provide moderate predictive efficacy for OS and PFS, the integrated model substantially enhances accuracy and reliability by consolidating multiple determinants.

## Discussion

The incidence and mortality of cervical cancer continue to increase, and treatment strategies are generally determined according to FIGO stage and anticipated prognosis. Nonetheless, variability in therapeutic approaches across medical institutions may influence survival outcomes. Accurate prognostic assessment in newly diagnosed patients enables more tailored therapeutic decisions, which is essential for extending survival and enhancing quality of life (Wang et al., 2021). Prognosis depends not only on tumor biology but also on host-related determinants, particularly nutritional status and immune-inflammatory responses. NPS, a composite scoring system integrating inflammatory and nutritional indices, has been applied for prognostic evaluation in tumors. PNI, as a representative nutritional parameter, has demonstrated prognostic relevance; however, its underlying mechanisms in predicting tumor stage, invasion, and lymph node metastasis remain incompletely elucidated, and evidence supporting its utility in gynecological malignancies remains limited. Comprehensive evaluation of the prognostic value and clinical applicability of PNI and NPS in cervical cancer is therefore warranted.

By retrospectively analyzing the clinical records of 465 cervical cancer patients, this study evaluated the prognostic relevance of the PNI and the NPS and further established a multifactorial prediction model. Both PNI and NPS, as objective nutritional-inflammatory indicators, showed strong associations with OS and PFS and contributed additional predictive strength within the integrated model, thereby providing a foundation for individualized prognostic assessment and clinical decision-making. Baseline analysis revealed that deceased patients presented with lower BMI, larger tumor burden, shorter diagnosis-to-treatment intervals, elevated tumor markers (SCC-Ag, CEA, CA125), and evidence of impaired nutritional status and heightened inflammatory activity, including reduced serum albumin, increased neutrophil counts, higher NLR, and decreased LMR and PNI. Results were consistent with prior studies, indicating that nutritional condition and inflammatory response exert significant influence on cervical cancer development and progression. Tumor growth and dissemination rely on host nutritional support and immune microenvironment, whereas chronic inflammation accelerates progression through mechanisms such as angiogenesis promotion and suppression of antitumor immunity, ultimately leading to adverse outcomes (Wang et al., 2022). For instance, previous evidence (Alwarawrah, Kiernan & MacIver, 2018) demonstrated that persistent inflammation induced epithelial-mesenchymal transition in tumor cells, thereby enhancing invasiveness and metastatic potential while simultaneously impairing immune surveillance, enabling tumor cells to evade immune-mediated eradication.

In addition, pathological analysis indicated that HPV-18 infection, adenocarcinoma histology, poor differentiation, advanced FIGO stage, and lymph node metastasis were more frequently observed in deceased patients. Survival outcomes were also inferior in patients who did not undergo surgery, radiotherapy, or chemotherapy. These observations reaffirm the prognostic relevance of conventional clinicopathological characteristics and treatment strategies in cervical cancer. HPV infection constitutes a key etiological factor, with subtype-specific oncogenic potential; HPV-18 in particular appears more prone to drive malignant progression compared with other variants (Wu et al., 2023). Adenocarcinoma, as a histological subtype, often exhibits more aggressive biological behavior and reduced treatment responsiveness, leading to unfavorable prognosis. Poorly differentiated tumors demonstrate higher malignancy, accelerated proliferation, and enhanced metastatic potential, accounting for the adverse outcomes in this subgroup. Advanced FIGO stage reflects extensive local invasion and dissemination, substantially increasing therapeutic challenges and limiting survival expectancy. Lymph node metastasis serves as a hallmark of disease advancement, enabling further dissemination of tumor cells through lymphatic pathways and promoting clinical deterioration. In contrast, active interventions such as surgery, radiotherapy, and chemotherapy contribute to effective tumor control and prolongation of survival (Xiao et al., 2022).

Kaplan–Meier survival analysis demonstrated that elevated LMR, reduced NLR, higher PNI, and lower NPS were consistently associated with prolonged OS and PFS, suggesting that nutritional-inflammatory indices provide effective stratification of prognostic risk in cervical cancer. Among these, PNI—reflecting both nutritional reserves and immune competence—emerged as particularly informative. Higher PNI levels indicate preserved nutritional capacity and intact immune function, supporting greater tolerance to tumor burden and treatment-induced injury (Komura et al., 2019). Consistent with this interpretation, multivariate analysis confirmed PNI as an independent protective factor for both OS and PFS. Adequate nutritional status ensures the energy and substrates required for normal metabolic processes and immune surveillance, thereby strengthening the capacity to identify and eradicate malignant cells. Moreover, sufficient nutrition mitigates treatment-related adverse effects from radiotherapy and chemotherapy, enhancing tolerance and adherence to therapeutic regimens (Nogueiro et al., 2022).

NPS quantifies the nutrition–inflammation equilibrium by integrating serum albumin, total cholesterol, NLR, and LMR. Lower NPS values reflect superior nutritional reserves and reduced inflammatory activity, which correspond to improved survival outcomes, emphasizing the prognostic relevance of the nutrition–inflammation axis in cervical cancer. Serum albumin serves as a sensitive marker of nutritional status, and reduced concentrations often signify malnutrition. Total cholesterol participates in diverse physiological functions, and abnormal levels may impair immune competence (Peng, Li & Li, 2022). NLR and LMR, representing the ratios of neutrophils to lymphocytes and lymphocytes to monocytes, respectively, provide indirect indicators of systemic inflammation and immune surveillance. Collectively, the combined assessment of these four indices yields a more accurate appraisal of the patient’s general condition and strengthens the reliability of prognostic evaluation. Cox regression analyses identified independent prognostic determinants for both OS and PFS. Multivariable analysis revealed that OS was independently influenced by BMI, CA125, PNI, HPV infection status, degree of differentiation, FIGO stage, and targeted therapy. Meanwhile, PFS was independently associated with BMI, tumor burden, PNI, HPV infection pattern, histological subtype, degree of differentiation, FIGO stage, and NPS. Notably, PNI retained statistical significance in both models, confirming its consistent role as a central prognostic marker.Our findings align with recent observations in other gynecological malignancies, reinforcing the utility of these markers across the field. For instance, a recent study by Tan & Chen (2023) on ovarian cancer demonstrated that low PNI was significantly associated with chemoresistance and poor survival, mirroring our results in cervical cancer. Similarly, Li et al. (2021) reported that NPS served as a robust prognostic stratifier in endometrial cancer, further validating the hypothesis that the nutrition-inflammation axis plays a universal role in driving tumor progression in gynecologic oncology. Prognostic implications of BMI were bidirectional: excessively low values often reflect malnutrition, with impaired immune capacity and diminished tumor resistance, whereas elevated BMI may correlate with chronic inflammatory states and endocrine disturbances that support tumor proliferation and dissemination.

CA125 represents a widely applied tumor marker, with elevated levels in cervical cancer frequently indicating disease progression and unfavorable prognosis (Chen et al., 2024). Persistent HPV infection, recognized as the principal etiological factor, induces abnormal epithelial proliferation and malignant transformation, while variations in HPV subtypes exert differential influences on patient outcomes (Polterauer et al., 2010). Traditional pathological parameters such as tumor differentiation and FIGO stage provide direct evidence of malignancy and disease advancement, thereby serving as essential determinants in prognostic assessment. In addition, targeted therapy, as an emerging therapeutic strategy, acts selectively on tumor-specific molecular targets, suppresses growth and metastasis, and contributes to prolongation of survival (Seebacher et al., 2019).

Analysis using the restricted cubic spline model identified an approximately linear inverse association between BMI and mortality risk for both OS and PFS, indicating that higher BMI was associated with better survival outcomes within the observed range. Conversely, PNI exhibited an inverse correlation with OS and PFS, suggesting that higher PNI levels were linked to reduced mortality risk and improved prognosis. These results provide a quantitative framework for nutritional interventions, supporting the formulation of individualized strategies based on BMI and PNI levels. Optimization of dietary composition, appropriate supplementation, and targeted adjustments to enhance nutritional status may improve PNI, thereby lowering mortality risk and extending survival. Building on the independent prognostic factors, nomogram models for OS and PFS were constructed and validated. Both models achieved high predictive accuracy for 3-, 5-, and 10-year survival, with calibration curves demonstrating close alignment between predicted and observed outcomes. Decision curve analysis confirmed substantial clinical net benefit, while time-dependent ROC analysis yielded AUC values consistently above 0.86, reflecting strong discriminative capacity. The nomogram, as a practical and comprehensible tool, integrates multiple prognostic variables to generate individualized survival estimates (Wang et al., 2025). By locating the scores corresponding to patient-specific indicators and summing them into a total score, clinicians can derive survival probabilities across defined timeframes, thereby informing more tailored therapeutic and follow-up strategies. The integration of these indices into routine decision-making pathways holds substantial clinical promise. Since PNI and NPS are derived from routine blood tests (albumin, cholesterol, and complete blood counts) that are mandatory for nearly all hospitalized patients, they offer a cost-effective and easily accessible method for pre-treatment risk stratification without additional burden. From an exploratory perspective, identifying high-risk patients based on our nomogram (*e.g.*, low PNI or high NPS) might eventually aid in triaging patients for multidisciplinary review. Future prospective studies are warranted to investigate whether preemptive nutritional interventions, such as immunonutrition support prior to surgery or chemoradiotherapy could potentially reverse the adverse inflammatory-nutritional status and improve therapeutic tolerance in these subgroups.

Compared with single-variable assessment, the integrated model achieved markedly higher predictive accuracy and reliability by incorporating BMI, tumor pathological characteristics, treatment strategies, and nutritional–inflammatory markers including PNI and NPS (Gou & Zhang, 2022). This outcome highlights the superiority of multifactorial evaluation in forecasting the prognosis of cervical cancer. The integration of conventional clinicopathological parameters with nutritional–inflammatory indicators enables a more comprehensive representation of the patient’s overall condition, thereby providing a stronger foundation for individualized therapeutic decision-making (Yang et al., 2022). While a solitary prognostic factor captures only a limited dimension of the patient’s status, multifactorial evaluation simultaneously accounts for nutritional status, inflammatory activity, tumor biology, and treatment modalities, yielding a more precise estimation of prognosis.

Subgroup analysis revealed significant associations between PNI, NPS, and prognosis across varying clinical characteristics and therapeutic regimens, albeit with notable heterogeneity (Gao et al., 2023). The protective effect of PNI was particularly evident in patients without diabetes, with squamous cell carcinoma, poor differentiation, or absence of lymph node metastasis, and significant interaction effects were observed with HPV infection status and histological type. A favorable prognosis was observed in the low NPS group among patients without comorbidities, with HPV16 infection, squamous cell carcinoma, or undergoing radiotherapy and chemotherapy (Fullerton et al., 2024), whereas the protective effect diminished in subgroups characterized by elevated NLR. These results indicate that the prognostic relevance of PNI and NPS is influenced by individual clinical profiles and treatment modalities. Consequently, these subgroup associations remain exploratory. Future studies with larger, independent cohorts are necessary to confirm whether these variations provide a reliable basis for refining risk stratification across different clinical phenotypes (Fullerton et al., 2024).

Diabetes, as a chronic metabolic disorder, impairs immune function and alters nutritional status, thereby diminishing the protective capacity of PNI. Variations in histological subtype and HPV infection status further influence tumor biology and therapeutic response, leading to heterogeneity in the prognostic relevance of PNI and NPS across subgroups. In addition, radiotherapy and chemotherapy can induce nutritional depletion and modulate systemic inflammation, which may alter the prognostic utility of NPS (Mohamud, Høgdall & Schnack, 2022). Within the high NLR subgroup, heightened inflammatory activity may obscure the protective effect of NPS, resulting in reduced prognostic discrimination.

The strengths of this study lie in its large sample size, extended follow-up, systematic assessment of multiple nutritional-inflammatory indices, and the development of a rigorously validated prediction model that can be applied in clinical settings. A sufficiently large cohort reduces random error and enhances the robustness of the conclusions, while prolonged follow-up provides a more accurate depiction of long-term survival outcomes. Furthermore, by comprehensively evaluating several nutritional-inflammatory indicators and identifying those with significant prognostic value, a reliable and integrative prediction model was established, offering clinicians a more precise and clinically meaningful tool for prognostic assessment.

Several limitations should be acknowledged in this study. First, the retrospective, single-center design inherently introduces selection bias and restricts the generalizability of our findings. Specifically, our reliance on a complete-case analysis by excluding patients with missing baseline laboratory data carries a high risk of selection bias, as these patients might systematically differ from those with complete records (*e.g.*, varying disease severities or different clinical triage pathways). This approach likely resulted in a more homogenous study cohort, which may have artificially inflated model performance measures, such as the reported AUC values. Consequently, the predictive accuracy of our nomograms should be interpreted with caution as a potentially overly optimistic estimation, and validation through multi-center prospective studies across diverse clinical settings is strictly required. Furthermore, a methodological source of potential optimism stems from our use of X-tile software to determine optimal cut-off values for nutritional-inflammatory indices within the same cohort used for model evaluation. Since these thresholds were data-driven and tailored to maximize survival differences in this specific dataset, the reported discriminative ability (*e.g.*, AUC values) may represent an upper bound of performance. Future studies employing independent training and validation sets are necessary to provide a more unbiased assessment of these cut-offs and the resulting model accuracy.Second, although the study period spans a decade, the 10-year long-term survival estimates are derived primarily from a subset of patients enrolled during the earlier phase of the study. Therefore, these long-term estimates must be interpreted cautiously due to variations in follow-up duration and the influence of censored data. Third, due to the retrospective data collection, certain hematological parameters, such as platelet counts, were not uniformly available for the full cohort, preventing the evaluation of other established prognostic markers like the Systemic Immune-Inflammation Index (SII). Future studies should incorporate a broader panel of inflammatory indices for a more comprehensive comparison. Fourth, a methodological limitation inherent to this study is the potential for multicollinearity among the evaluated nutritional-inflammatory indices. Because indices such as PNI, NPS, NLR, and LMR share foundational physiological parameters (*e.g.*, serum albumin and lymphocyte counts), they inevitably reflect overlapping biological pathways. Although we deliberately excluded single constituent biomarkers during variable selection to maintain statistical stability, the inclusion of these interrelated composite indices in the same analytical framework should be noted. Therefore, the independent prognostic weights assigned to these markers in our multivariable models should be interpreted carefully as exploratory representations of relative risk. Finally, measurement bias cannot be entirely excluded. Despite standardized data collection, retrospective laboratory records remain susceptible to variations in detection methods, equipment, and operator performance over time. Moreover, this study did not address the underlying molecular mechanisms linking PNI and NPS with cervical cancer prognosis. Future investigations integrating basic research are essential to elucidate these mechanistic pathways, which could provide a theoretical framework for novel therapeutic targets and intervention strategies.

## Conclusions

This study suggests that PNI may serve as a potential independent prognostic factor for both OS and PFS in this cohort of cervical cancer patients. While NPS was identified as an independent predictor specifically for PFS, it also showed a trend toward risk stratification for OS in univariate analysis. As readily obtainable nutritional-inflammatory indicators, both indices may provide preliminary insights that complement conventional clinicopathological parameters. The prognostic nomograms developed in this study integrate these markers with key clinical features to generate individualized survival hypotheses. Given the retrospective design, the potential for optimism bias due to internal optimization of cut-off values, and the lack of external validation, these predictive models and subgroup analyses should be framed strictly as exploratory and hypothesis-generating. While they offer a preliminary conceptual framework, the clinical utility and discriminative performance of these tools remain unproven. Extensive external validation in diverse independent populations is essential before these preliminary findings can be considered for routine clinical decision-making or for guiding preemptive nutritional interventions. Future research should aim to implement multi-center prospective validation to assess the generalizability of these models and investigate the molecular pathways linking nutritional-inflammatory status to cervical cancer progression. Ultimately, further investigation into assessing and optimizing the nutrition-inflammation axis is warranted to explore its role in enhancing immune competence and improving long-term survival in cervical cancer.

## Supplemental Information

10.7717/peerj.21273/supp-1 Supplemental Information 1 Univariate and multivariate COX regression analysis of overall survival in patients with cervical cancer The results of univariate and multivariate COX regression analyses for overall survival (OS) in patients with cervical cancer. Abbreviations: OS, overall survival; HR, hazard ratio; 95% CI, 95% confidence interval; *P*, *P* value. HR > 1 indicates an increased risk of death, while HR < 1 indicates a decreased risk. Variables with *P* < 0.05 in univariate analysis were included in the multivariate COX regression analysis to identify independent prognostic factors for OS. 1 indicates an increased risk of death, while HR < 1 indicates a decreased risk. Variables with *P*< 0.05 in univariate analysis were included in the multivariate COX regression analysis to identify independent prognostic factors for OS.

10.7717/peerj.21273/supp-2 Supplemental Information 2 Univariate and multivariate COX regression analysis of progression-free survival (PFS) in patients with cervical cancer The results of univariate and multivariate COX regression analyses for progression-free survival (PFS) in patients with cervical cancer. Abbreviations: PFS, progression-free survival; HR, hazard ratio; 95%CI, 95% confidence interval; *P*, *P* value. HR > 1 indicates an increased risk of disease progression or death, while HR < 1 indicates a decreased risk. Variables with *P* < 0.05 in univariate analysis were included in the multivariate COX regression analysis to identify independent prognostic factors for PFS. 1 indicates an increased risk of disease progression or death, while HR < 1 indicates a decreased risk. Variables with *P* < 0.05 in univariate analysis were included in the multivariate COX regression analysis to identify independent prognostic factors for PFS.
Variables include demographics, tumour characteristics, treatment modalities, nutritional-inflammatory indices (NLR, LMR, PNI, NPS) and follow-up outcomes (PFS, OS). All personal identifiers were removed; only study-specific codes are retained.

10.7717/peerj.21273/supp-3 Supplemental Information 3 Baseline Characteristics and Nutritional-Inflammatory Indices of 465 Cervical Cancer Patients (Anonymized Raw Dataset) Raw data file containing anonymized clinical, laboratory and survival variables for 465 cervical cancer patients (CSV format); variables include demographics, tumour characteristics, treatment modalities, nutritional-inflammatory indices (NLR, LMR, PNI, NPS) and follow-up outcomes (PFS, OS).

10.7717/peerj.21273/supp-4 Supplemental Information 4 Digit-to-Label Mappings of Categorical Variables and Cut-off Criteria for NLR, LMR, PNI and NPS Biomarkers Digit-to-label mappings for all categorical variables used in the raw dataset (*e.g.*, FIGO_Stage: 1 = Grade I, 2 = Grade II, 3 = Grade III, 4 = Grade IV), along with tertiary cut-offs for NLR, LMR, PNI and binary labels for NPS.

## Additional Information and Declarations

### Data Availability

The following information was supplied regarding data availability: The anonymized clinical, laboratory and survival variables for 465 cervical cancer patients are available in the Supplemental File.
