# Supplementary material for: A retrospective analysis of the prognostic value of nutritional-inflammatory markers for patients with cervical cancer
Source: PeerJ. 2026 May 6;14:e21273. doi: 10.7717/peerj.21273 (PMC13156955; doi:10.7717/peerj.21273)
Supplement: Supplemental Information 1 — The results of univariate and multivariate COX regression analyses for overall survival (OS) in patients with cervical cancer. Abbreviations: OS, overall survival; HR, hazard ratio; 95% CI, 95% confidence interval; P, P value. HR > 1 indicates an increased risk of death, while HR < 1 indicates a decreased risk. Variables with P < 0.05 in univariate analysis were included in the multivariate COX regression analysis to identify independent prognostic factors for OS. 1 indicates an increased risk of death, while HR < 1 indicates a decreased risk. Variables with P< 0.05 in univariate analysis were included in the multivariate COX regression analysis to identify independent prognostic factors for OS. [file peerj-14-21273-s001.docx]

Table S1:Univariate and multivariate COX regression analysis of overall survival in patients with cervical cancer

| Variables | **Univariate analysis** | *p* | **Multivariate analysis** | *p* |
| --- | --- | --- | --- | --- |
|  | **HR (95%CI)** |  | **HR (95%CI)** |  |
| Age, years | 1.004(0.985,1.023) | 0.684 | - | - |
| BMI | 0.870(0.805,0.941) | <0.001 | **0.901(0.837,0.970)** | **0.005** |
| Tumor_size | 1.630(1.473,1.804) | <0.001 | **1.150(1.004,1.318)** | **0.044** |
| Diagnosis_to_Treatment_Interval | 1.000(0.998,1.002) | 0.945 | - | - |
| SCC_Ag | 1.037(1.024,1.051) | <0.001 | 1.016(0.995,1.038) | 0.125 |
| CEA | 1.027(1.013,1.041) | <0.001 | **1.020(1.001,1.040)** | **0.037** |
| CA125 | 1.003(1.001,1.004) | <0.001 | **1.002(1.001,1.003)** | **0.002** |
| Albumin | 0.853(0.816,0.892) | <0.001 | - | - |
| Total_Cholesterol | 0.864(0.697,1.071) | 0.181 | - | - |
| Lymphocyte | 0.862(0.590,1.261) | 0.445 | - | - |
| Neutrophil | 1.209(1.133,1.291) | <0.001 | - | - |
| Monocyte | 1.278(1.028,1.588) | 0.027 | - | - |
| NLR | 1.149(1.097,1.203) | <0.001 | - | - |
| LMR | 0.842(0.762,0.930) | <0.001 | - | - |
| PNI | 0.890(0.857,0.924) | <0.001 | - | - |
| Diabetes, n(%) |  |  |  |  |
| No | Reference |  |  |  |
| Yes | 1.610(0.859,3.017) | 0.137 | - | - |
| Hypertension, n(%) |  |  |  |  |
| No | Reference |  |  |  |
| Yes | 1.211(0.733,2.002) | 0.454 | - | - |
| HPV_Infection, n(%) |  |  |  |  |
| 16 | Reference |  | Reference |  |
| 18 | 2.964(1.633,5.378) | <0.001 | **4.400(1.926,10.055)** | **<0.001** |
| Others | 0.839(0.454,1.548) | 0.574 | 1.346(0.699,2.591) | 0.374 |
| Negative | 1.969(1.177,3.292) | 0.010 | 1.154(0.638,2.088) | 0.636 |
| Histology_type, n(%) |  |  |  |  |
| Squamous cell carcinoma | Reference |  | Reference |  |
| Adenocarcinoma | 2.689(1.656,4.365) | <0.001 | 1.558(0.763,3.184) | 0.224 |
| Degree_of_differentiation, n(%) |  |  |  |  |
| Low-differentiated | Reference |  | Reference |  |
| Medium-differentiated | 0.206(0.128,0.330) | <0.001 | **0.431(0.249,0.747)** | **0.003** |
| High-differentiated | 0.079(0.019,0.323) | <0.001 | 0.319(0.071,1.433) | 0.136 |
| FIGO_Stage, n(%) |  |  |  |  |
| Grade I | Reference |  | **Reference** |  |
| Grade II | 1.472(1.167,2.335) | <0.001 | **1.271(1.025,2.206)** | **0.004** |
| Grade III | 2.190(1.604,4.049) | <0.001 | **2.040(1.285,4.312)** | **0.001** |
| Grade IV | 3.524(2.346,6.908) | <0.001 | **3.157(2.114,6.379)** | **<0.001** |
| Lymph_node_metastasis, n(%) |  |  |  |  |
| No | Reference |  | Reference |  |
| Yes | 5.789(3.814,8.787) | <0.001 | 1.247(0.653,2.384) | 0.503 |
| Radiotherapy, n(%) |  |  |  |  |
| No | Reference |  | Reference |  |
| Yes | 5.816(2.545,13.294) | <0.001 | 1.442(0.549,3.790) | 0.458 |
| Chemotherapy, n(%) |  |  |  |  |
| No | Reference |  | Reference |  |
| Yes | 7.594(3.322,17.358) | <0.001 | 0.891(0.334,2.377) | 0.818 |
| Surgery, n(%) |  |  |  |  |
| No | Reference |  | Reference |  |
| Yes | 0.173(0.113,0.265) | <0.001 | 0.807(0.474,1.375) | 0.431 |
| Targeted_therapy, n(%) |  |  |  |  |
| No | Reference |  | Reference |  |
| Yes | 2.122(1.263,3.564) | 0.004 | **0.376(0.189,0.746)** | **0.005** |
| NLR, n(%) |  |  |  |  |
| <2.9 | Reference |  | Reference |  |
| 2.9-4.8 | 1.856(1.175,2.933) | 0.008 | 1.015(0.563,1.830) | 0.959 |
| >4.8 | 3.101(1.842,5.220) | <0.001 | 0.697(0.317,1.536) | 0.371 |
| LMR, n(%) |  |  |  |  |
| <2.7 | Reference |  | Reference |  |
| 2.7-3.8 | 0.563(0.325,0.975) | 0.040 | 0.623(0.311,1.248) | 0.182 |
| >3.8 | 0.297(0.182,0.485) | <0.001 | 0.785(0.396,1.555) | 0.487 |
| PNI, n(%) |  |  |  |  |
| <46.5 | Reference |  | Reference |  |
| 46.5-51.8 | 0.385(0.235,0.630) | <0.001 | **0.615(0.346,0.991)** | **0.046** |
| >51.8 | 0.194(0.114,0.330) | <0.001 | **0.430(0.211,0.878)** | **0.020** |
| NPS, n(%) |  |  |  |  |
| L_NPS | Reference |  | Reference |  |
| H_NPS | 1.419(1.275,2.636) | <0.001 | 1.066(0.538,2.113) | 0.855 |
