# Supplementary material for: A retrospective analysis of the prognostic value of nutritional-inflammatory markers for patients with cervical cancer
Source: PeerJ. 2026 May 6;14:e21273. doi: 10.7717/peerj.21273 (PMC13156955; doi:10.7717/peerj.21273)
Supplement: Supplemental Information 2 — The results of univariate and multivariate COX regression analyses for progression-free survival (PFS) in patients with cervical cancer. Abbreviations: PFS, progression-free survival; HR, hazard ratio; 95%CI, 95% confidence interval; P, P value. HR > 1 indicates an increased risk of disease progression or death, while HR < 1 indicates a decreased risk. Variables with P < 0.05 in univariate analysis were included in the multivariate COX regression analysis to identify independent prognostic factors for PFS. 1 indicates an increased risk of disease progression or death, while HR < 1 indicates a decreased risk. Variables with P < 0.05 in univariate analysis were included in the multivariate COX regression analysis to identify independent prognostic factors for PFS. Variables include demographics, tumour characteristics, treatment modalities, nutritional-inflammatory indices (NLR, LMR, PNI, NPS) and follow-up outcomes (PFS, OS). All personal identifiers were removed; only study-specific codes are retained. [file peerj-14-21273-s002.docx]

Table S2:Univariate and multivariate COX regression analysis of PFS in patients with cervical cancer

| Variables | **Univariate analysis** | *p* | **Multivariate analysis** | *p* |  |
| --- | --- | --- | --- | --- | --- |
|  | **HR (95%CI)** |  | **HR (95%CI)** |  |  |
| Age, years | | 1.005(0.988,1.022) | 0.579 | - | - |
| BMI | | **0.877(0.820,0.939)** | **<0.001** | **0.904(0.848,0.964)** | **0.002** |
| Tumor_size | | **1.593(1.458,1.740)** | **<0.001** | **1.179(1.042,1.333)** | **0.009** |
| Diagnosis_to_Treatment_Interval | | 1.000(0.999,1.001) | 1.000 | - | - |
| SCC_Ag | 1.041(1.029,1.053) | <0.001 | 1.013(0.995,1.031) | 0.154 |  |
| CEA | 1.024(1.010,1.038) | <0.001 | 0.999(0.979,1.019) | 0.893 |  |
| CA125 | 1.003(1.002,1.004) | <0.001 | 0.999(0.997,1.001) | 0.188 |  |
| Albumin | 0.861(0.825,0.898) | <0.001 | - | - |  |
| Total_Cholesterol | 0.936(0.777,1.128) | 0.486 | - | - |  |
| Lymphocyte | 0.934(0.667,1.307) | 0.692 | - | - |  |
| Neutrophil | 1.225(1.157,1.298) | <0.001 | - | - |  |
| Monocyte | 1.276(1.053,1.547) | 0.013 | - | - |  |
| NLR | 1.117(1.080,1.157) | <0.001 | - | - |  |
| LMR | 0.828(0.756,0.907) | <0.001 | - | - |  |
| PNI | 0.902(0.871,0.935) | <0.001 | - | - |  |
| Diabetes, n(%) |  |  |  |  |  |
| No | Reference |  |  |  |  |
| Yes | 1.738(0.995,3.036) | 0.052 | - | - |  |
| Hypertension, n(%) |  |  |  |  |  |
| No | Reference |  |  |  |  |
| Yes | 1.388(0.899,2.143) | 0.138 | - | - |  |
| HPV_Infection, n(%) |  |  |  |  |  |
| 16 | Reference |  | Reference |  |  |
| 18 | 2.209(1.239,3.937) | 0.007 | **1.933(1.371,3.290)** | **0.025** |  |
| Others | 0.698(0.398,1.224) | 0.210 | 1.021(0.562,1.856) | 0.944 |  |
| Negative | 1.777(1.134,2.786) | 0.012 | 1.048(0.635,1.730) | 0.854 |  |
| Histology_type, n(%) |  |  |  |  |  |
| Squamous cell carcinoma | Reference |  | **Reference** |  |  |
| Adenocarcinoma | 2.309(1.465,3.638) | <0.001 | **2.312(1.210,4.415)** | **0.011** |  |
| Degree_of_differentiation, n(%) |  |  |  |  |  |
| Low-differentiated | Reference |  | Reference |  |  |
| Medium-differentiated | 0.199(0.131,0.302) | <0.001 | **0.390(0.242,0.627)** | **<0.001** |  |
| High-differentiated | 0.086(0.027,0.273) | <0.001 | 0.335(0.099,1.138) | 0.080 |  |
| FIGO_Stage, n(%) |  |  |  |  |  |
| Grade I | Reference |  | **Reference** |  |  |
| Grade II | 1.258(1.029,2.873) | <0.001 | **1.187(1.014,2.340)** | **0.004** |  |
| Grade III | 2.786(1.645,4.932) | <0.001 | **2.286(1.529,4.536)** | **<0.001** |  |
| Grade IV | 3.710(2.158,6.208) | <0.001 | **3.359(2.227,6.150)** | **<0.001** |  |
| Lymph_node_metastasis, n(%) |  |  |  |  |  |
| No | Reference |  | Reference |  |  |
| Yes | 5.968(4.120,8.644) | <0.001 | 1.223(0.690,2.167) | 0.490 |  |
| Radiotherapy, n(%) |  |  |  |  |  |
| No | Reference |  | Reference |  |  |
| Yes | 5.009(2.539,9.882) | <0.001 | 1.330(0.576,3.070) | 0.505 |  |
| Chemotherapy, n(%) |  |  |  |  |  |
| No | Reference |  | Reference |  |  |
| Yes | 5.938(3.108,11.347) | <0.001 | 0.847(0.381,1.883) | 0.683 |  |
| Surgery, n(%) |  |  |  |  |  |
| No | Reference |  | Reference |  |  |
| Yes | 0.193(0.133,0.280) | <0.001 | 0.893(0.551,1.446) | 0.645 |  |
| Targeted_therapy, n(%) |  |  |  |  |  |
| No | Reference |  | Reference |  |  |
| Yes | 3.564(2.359,5.384) | <0.001 | 1.001(0.592,1.694) | 0.996 |  |
| NLR, n(%) |  |  |  |  |  |
| <2.9 | Reference |  | Reference |  |  |
| 2.9-4.8 | 1.916(1.280,2.869) | 0.002 | 0.980(0.588,1.632) | 0.937 |  |
| >4.8 | 3.015(1.879,4.838) | <0.001 | 0.763(0.365,1.597) | 0.473 |  |
| LMR, n(%) |  |  |  |  |  |
| <2.7 | Reference |  | Reference |  |  |
| 2.7-3.8 | 0.578(0.356,0.937) | 0.026 | 0.621(0.340,1.135) | 0.122 |  |
| >3.8 | 0.278(0.179,0.433) | <0.001 | 0.659(0.352,1.233) | 0.192 |  |
| PNI, n(%) |  |  |  |  |  |
| <46.5 | Reference |  | **Reference** |  |  |
| 46.5-51.8 | 0.433(0.274,0.684) | <0.001 | **0.760(0.504,0.965)** | **0.013** |  |
| >51.8 | 0.236(0.146,0.380) | <0.001 | **0.688(0.416,0.992)** | **0.024** |  |
| NPS, n(%) |  |  |  |  |  |
| L_NPS | Reference |  | **Reference** |  |  |
| H_NPS | 1.423(1.192,2.613) | <0.001 | **1.301(1.090,2.122)** | **0.007** |  |
